# Acquired Hemophilia presenting as Gross hematuria following Kidney Stone – A case report and review of the literature

**DOI:** 10.1590/S1677-5538.IBJU.2017.0172

**Published:** 2018

**Authors:** Max Schmidt-Bowman, Lael Reinstatler, Eric P. Raffin, Joseph E. Yared, John D. Seigne, Einar F. Sverrisson

**Affiliations:** 1 Geisel School of Medicine at Dartmouth, Hanover, NH; 2 Department of Surgery, Section of Urology, Dartmouth Hitchcock Medical Center, Lebanon, NH

**Keywords:** Hematuria, Nephrolithiasis, Factor 8 deficiency, acquired [Supplementary Concept]

## Abstract

A rare condition in itself, acquired hemophilia A, seldom presents as isolated gross hematuria. It is a serious condition with a high mortality rate and thus clinical suspicion followed by prompt diagnosis is imperative (1). In fact, only 8 cases of such presentation of this condition have been reported thus far in the literature. Of these, none describe the initial presentation of hematuria with the inciting event of a kidney stone. We present a case of a 67-year-old man with signs and symptoms of nephrolithiasis accompanied by profuse hematuria, who was subsequently found to have developed expression of factor VIII inhibitor leading to acquired hemophilia A.

## CASE REPORT

A 67-year-old man was transferred to our hospital, a large academic medical center, for work-up of persistent gross hematuria. The patient first presented to his local hospital eight days prior with the complaint of gross hematuria and left flank pain. A computed tomography (CT) scan was obtained, revealing an obstructing 2-3 mm left ureteropelvic junction (UPJ) stone and associated hydronephrosis. Physical exam also showed a fever of 102F. Urologic evaluation was not available so he was subsequently transferred to a different outside hospital for management, where he was admitted to the medical intensive care unit (MICU) and taken to the operating room (OR) for ureteral stent placement. A retrograde pyelogram was normal without filling defects, however urine from his left ureteral orifice was noted to be bloody and there was a hydronephrotic drip reported. A postoperative abdominal and pelvic CT with and without contrast showed clot in the left collecting system and hydronephrosis with a 3 mm lower pole stone. His hematuria persisted, though his vital signs and hemoglobin were stable. He was ultimately discharged home on hospital day 5 with an indwelling Foley catheter and plans to follow-up with his primary urologist. He returned to his primary urologist several days later with persistent gross hematuria and passing clots through his Foley catheter, for which continuous bladder irrigation (CBI) therapy was initiated. His hemoglobin was noted to have fallen to 8.6mg/dL from 10.8mg/dL in the past week. At this time, he was transferred to our hospital for further work up and management.

Notable past medical history for this patient includes acute lymphocytic leukemia (ALL) diagnosed 4 years prior to this current admission, for which he received an allo-stem cell transplant. He subsequently developed graft versus host disease (GVHD), but otherwise did well and maintained his immunosuppression on mycophenolate and prednisone without any issues. Labs on presentation were normal except for hemoglobin of 8.5mg/dL and PTT of 98 (normal range 25 - 35 seconds). His WBC was 6.2, platelet count was 217, creatinine was 1.05 mg/dL, PT was 15.4 (normal range 12 - 15 seconds) and INR was 1.2. Given his elevated PTT the Hematology service was consulted. They ordered further blood work that demonstrated normal fibrinogen and thrombin time levels. On mixing studies, he was found to have a Factor 8 inhibitor level of 384 (normal range 0.0 - 0.4).

Our patient was subsequently diagnosed with acquired hemophilia A and transferred to the hematology service for further management. He continued on CBI and conservative management for his hematuria. Therapy was initiated with Novo-7 and FEIBA (activated prothrombin complex concentrate or aPCC, dosed at 70 units/kg) daily until resolution of hematuria, and five weekly doses of Rituximab. During his hospitalization, he required one transfusion of pRBCs. His hematuria improved enough that his CBI was stopped and then his Foley catheter was removed. He was discharged home with continued follow-up and infusions per the Hematology team.

## DISCUSSION

Acquired hemophilia A represents a rare diagnosis with an incidence of about 1/1,000,000 and mortality rates as high as 1/5 (1-3). Presentation is varied and the condition is often misdiagnosed until a thorough hematologic evaluation takes place. An early indicator and finding that should prompt hematologic referral is the finding of an isolated elevated PTT. Autoimmune hemophilia is caused by inhibitory auto-antibodies against a coagulation factor, most often factor 8 (4). An associated illness such as malignancy, auto-immune diseases, or certain medications are only involved half of the time; the remaining cases are idiopathic. This makes the diagnosis even more challenging.

The initial clinical presentation for acquired hemophilia is usually easy bruising and soft tissue bleeding. Retroperitoneal bleeding has also been described as an initial presentation; these cases have high mortality rates as the diagnosis is usually missed and the bleeding is difficult to control (3). In our case, after further questioning, our patient did reveal that he bit the inside of his mouth before the gross hematuria episode and this took a week to heal. He also recently had a nevus removed from his forearm and this bled for about a week as well.

Acquired hemophilia presenting as gross hematuria is not altogether unheard of, as demonstrated by two case reports in the literature. Shander et al. describe an 82-year-old male who presented with 8 days of gross hematuria. On further work up, he was determined to have acquired hemophilia A with subsequent spontaneous hemorrhage of a previously identified renal cyst (5). Similarly, Hosier et al. report the case of a 54-year-old female who presented with gross hematuria and was later diagnosed with acquired hemophilia A. In this case, however, the inciting cause of bleeding was not identified and it was assumed to be secondary to spontaneous hemorrhage (6). A third case describes gross hematuria in a 60-year-old male with congenital hemophilia A rather than acquired hemophilia A, however the case is notable because the genitourinary hemorrhage was so profound that a cystectomy with ileal conduit was eventually required (7). Urolithiasis has not previously been reported as the inciting cause of hematuria in acquired hemophilia A, as was the case in the patient described above.

Various agents have been used to acutely treat this disorder with success mostly related to the severity of the bleed. Patients with low auto-antibody titers may be treated with human factor VIII concentrate and DDAVP (1-deamino-8-D-arginine vasopressin) whereas patients with high titers are usually treated with aPCC (FEIBA) (8). In their review of fourteen patients with acquired hemophilia over 8 years, Holme et al. describe their success using FEIBA, NovoSeven, corticosteroids, and/or cyclophosphamide. Two of their patients had no response to treatment, six had a partial response, and the remainder were successfully treated (1). Importantly, they noted that some of the cohort required procedures and these patients were effectively treated with FEIBA peri-operatively without any bleeding complications. In a larger retrospective review, Lak et al described their experience with 34 patients diagnosed with acquired hemophilia A. Over 79% of their patients experienced complete response to treatment. The median time of treatment was 4 months. Seven of their patients required surgical procedures and all were effectively treated without any intraoperative bleeding complications. Two of their patients died, one from bleeding and one from their underlying disease (2).

## CONCLUSION

Acquired hemophilia A is a rare bleeding disorder that may present as gross hematuria, with or without an inciting factor such as urolithiasis. This diagnosis should be suspected in the setting of an isolated prolonged PTT. Prompt recognition and treatment can be lifesaving.
